# What do rates of deposition of dental cementum tell us? Functional and evolutionary hypotheses in red deer

**DOI:** 10.1371/journal.pone.0231957

**Published:** 2020-04-28

**Authors:** F. J. Pérez-Barbería, F. E. Guinness, M. López-Quintanilla, A. J. García, L. Gallego, J. Cappelli, M. P. Serrano, T. Landete-Castillejos

**Affiliations:** 1 Game and Livestock Resources Unit, University of Castilla-La Mancha, IDR, IREC, Albacete, Spain; 2 Wildlife Research Unit UIRCP, Universidad de Córdoba, Córdoba, Spain; 3 Department of Zoology, Large Animal Research Group, University of Cambridge, Cambridge, United Kingdom; Università degli Studi della Campania, ITALY

## Abstract

Cementum is a bone connective tissue that provides a flexible attachment for the tooth to the alveolar bone in many mammalian species. It does not undergo continuous remodelling, unlike non-dental bone, which combined with its growth pattern of seasonal layering makes this tissue uniquely suitable as a proxy for tracking changes in body repair investment throughout an animal´s life. We tested functional and sexual selection hypotheses on the rate of cementum deposition related to the highly polygynous mating strategy of red deer. We used a sample of 156 first lower molars from wild Scottish red deer of known age between 1 and 17 years old, approximately balanced by sex and age class. Cementum deposition on the inter-radicular pad increased with age at a constant average rate of 0.26 mm per year, with no significant differences between sexes. Cementum deposition was independent of (i) tooth wear, other than that associated with age, and (ii) enamel and dentine micro-hardness. The results partially supported the hypothesis that the main function of cementum is the repositioning of the tooth to maintain opposing teeth in occlusion. However, teeth that had more wear or males´ teeth that had faster rates of tooth wear than those of females did not present the expected higher rates of cementum deposition.

## Introduction

Cementum is a dynamic connective dental bone tissue that provides a flexible attachment structure via the periodontal ligament in mammals and crocodilians. Recent studies indicate that cementum and periodontal ligament are plesiomorphic traits in Amniota [1]. Cementum is mainly deposited on the radicular dentine of the root apex and on the furcations of multi-rooted teeth, forming an inter-radicular pad [2], although the distribution varies with species, and many mammals (e.g. ungulates, elephants, rodents, odontocete whales) have extensive coronal cement coatings [3]. Mammalian cementum is unique in that it is avascular [although it can be vascular in some reptilians [4]] receiving its nutrition through embedded cells (cementocytes) that feed from the vascular periodontal ligament. Cementum does not undergo continuous remodelling under normal circumstances, unlike non-dental bone, but continues to grow in thickness throughout life [3]. Its growth pattern of seasonal layering, resulting from variations in microstructure [5], has been extensively used in archaeology, life history studies in population ecology [6–10] and as a useful technique to estimate age [11–13]. However, there is a lack of information on the rate of cementum deposition over an animal´s life and its functional, ecological and evolutionary significance in ungulates. This is due to the fact that the functional mechanisms that drive the activity of the cementoblasts remain obscure.

Cementum is composed of equal parts per volume of water, organic matrix and mineral [2,3]. About 50% of the dry mass is an organic matrix containing mainly collagen fibres embedded in an interfibrillar ground substance of glycoproteins. About 90% of collagen is type I and 5% is type III, with the remaining 5% being glycosaminoglycans, chondroitin 4-sulphate, dermatan sulphate, and non-collagenous proteins such as alkaline phosphatase. The other 50% of the dry mass is inorganic, mainly calcium and phosphate in the form of hydroxyapatite crystals, and traces of the elements copper, fluorine, iron, lead, potassium, silicon, sodium and zinc [2,3].

Primary cementum is laid by cementoblasts situated on the surface of the dentine, where they make a layer of acellular cementum around the cervical part of the root before the tooth reaches the occlusal plane. Acellular cementum is mainly formed by Sharpey´s fibres (extrinsic fibres), which come from the periodontal ligament. They are inserted perpendicular to the root surface, where primary cementum is mineralised with thin flakes of hydroxyapatite at such a fast rate of deposition that the incremental lines are wide apart.

The secondary cementum develops mainly on the apical portion of the root in mammals, when the tooth reaches the occlusal plane. It contains cementocytes that are trapped in individual lacunae and is less mineralised than the acellular cementum, although the hydroxyapatite crystals are larger and globular. There are less Sharpey´s fibres and other fibres derived from the cementoblasts (intrinsic fibres) run parallel to the root surface. There is also an area of mixed fibre cementum where intrinsic and periodontal ligament fibres meet.

Changes in cementum microstructure are responsible for its layering structure, both around the root apex and root furcation in multi-rooted teeth. There are two main mechanisms that affect the microstructure of cementum, (i) changes in the rate of tissue growth together with differences in composition and degree of mineralization [3,14], and (ii) variation in the orientation of the fibres [5]. Slow deposition of mixed fibre cementum, poor in intrinsic fibres and cells yields thin layers. Thicker and more irregular cementum layers are produced at faster rates of deposition, are richer in intrinsic fibres and entrap more extrinsic fibres and cells. Tooth occlusal surfaces are repositioned by resorption of the extrinsic fibres in the periodontal ligament, and new fibres are entrapped by growing pre-cementum [3]. The pattern of tooth reposition can be tracked by following changes in the orientation of fibres [15].

The cause of the seasonal pattern in cementum deposition is not yet clear. It has been suggested that it might be related to seasonal changes in diet composition or other aspects of physiology. Saxon and Higham [16] suggested that a decrease of fodder availability during winter might be responsible for less tooth wear and so less compensatory deposition of cementum. This would not apply equally to wild and domestic species, as the latter are less subject to dietary changes. The cementum layering pattern has been used as a convenient trait to estimate the age in many population studies [8–11,13], but it also inspires research on the biological, ecological and evolutionary mechanisms that might drive different rates of cementum deposition, which is the aim of this study.

The disposable soma theory of senescence postulates that the durability of somatic structures should correlate positively with reproductive lifespan [17]. In highly polygynous species, in which females have very limited number of offspring per birth, it has been demonstrated that females live longer than males to maximise their reproductive output. Males instead invest in reproduction during their prime when success for mating opportunities via male-male competition is at its highest [18]. In these species there is evidence that males wear their teeth faster than their conspecific females [9,19–22] and that males´ tooth size is smaller than in females, in agreement with the disposable soma theory [23]. If cementum is a flexible mechanism of tooth attachment to the alveolus and a structure to enable repositioning of the occlusal plane, then, the rate at which cementum is deposited could be used as an evolutionary life history trait, something that has never been investigated.

In this paper we test the following hypotheses on the rate of cementum deposition on the root furcation pad of the first lower molar of Scottish red deer (Table 1): H1. If the main function of cementum is the repositioning of the tooth to maintain opposing teeth in occlusion and adjacent teeth in mesial-distal contact, then, the cementum root pad should get thicker as the animal gets older; H2. In addition, for animals of the same age and size those that wear their teeth faster should present thicker cementum pads than animals with lower rates of tooth wear; H3. There is evidence that red deer males wear their teeth faster than their conspecific females (see above), consequently, in agreement with H1 males should develop thicker cementum pads than females after controlling for tooth size and age; H4. It has been hypothesised that males of polygynous species reduce their investment in somatic maintenance after they pass their reproductive prime, while females of the same species sustain a constant investment in maintenance to maximise their reproductive fitness across their life. Consequently, it should be expected that the rate of cementum deposition slows down in males after their prime in comparison with those in females; H5. Red deer stags reduce their feeding activity during the rutting season and it has been suggested that this decreases the rate of cementum deposition [12]. As a consequence the radicular pad thickness should increase at a slower rate in males after they become reproductively active than in females of similar age; H6. Harder teeth wear more slowly than softer teeth under similar conditions of mastication effort and diet, therefore and in agreement with H2, harder teeth should present thinner inter-radicular cementum pads than softer teeth; H7. The prediction in H6 would not hold if hardness of dental tissue changes across the life of an animal, as happens with dentine, which hardens as the animal gets older by a process of mineralisation [24,25], while enamel hardness mineralisation is not so age-dependent [26]; H8. If cementum deposition functions as a repair mechanism then higher deposition rates should be expected during times when teeth suffer the most stress. That is at periods of maximum intake rates, during growth and prime condition, but also at old age when tooth wear by mesial attrition creates diastemata between adjacent teeth, leaving teeth less supported and so exposed to higher labial-buccal and mesial-distal movement stress.

**Table 1:** Hypotheses and predictions on cementum deposition rate across life in red deer. Type of hypothesis: F, functional; S, sexual selection.

Hypothesis	Type	Prediction
H1. Maintenance of tooth in occlusion	F	Cementum deposition increases with age
H2. Maintenance of tooth in occlusion	F	Cementum deposition increases with tooth wear
H3. Sexual selection in tooth wear	S	Higher rate of cementum deposition in males than in females
H4. Sexual selection in somatic repair	S	Cementum deposition decreases in males after prime age
H5. Effect of rut in mastication activity	S	Cementum deposition decreases in males during rut
H6. Effect of tooth hardness	F	Cementum deposition rate is lower in harder teeth
H7. Tooth hardness is affected by tooth mineralisation with age	F	H6 prediction does not hold when controlling for age
H8. Repair mechanism	F	Cementum deposition rate higher at the periods of maximum mastication activity and dental stress

## Materials and methods

### Tooth sampling

This study used the red deer teeth sample from Pérez-Barbería et al [11] and Pérez-Barbería [27], which is summarised by cohort, sex and age in Tables 2 and 3. It is comprised of 156 first lower molars (M_1_) of wild Scottish red deer of known age, between 1 and 17 years old, born between 1980 and 2006 on the island of Rum (Scotland). The teeth came from animals that were individually identified using ear tags, their age was accurate to within a month. The sample was approximately balanced by sex and age class (Table 3).

**Table 2:** Number of red deer of known age by sex and cohort. Cohort is coded by the last two digits of the year, between 1980 and 2006.

Cohort	80	83	84	85	86	87	88	89	90	91	92	93	94	95	96	97	98	99	00	01	02	03	05	06
female	1	1	4	3	3	2	2	4	2	2	3	6	9	7	4	7	9	6	4	1	0	3	2	1
male	0	0	1	3	4	2	1	1	5	2	1	3	6	12	7	1	4	3	2	2	3	7	0	0

**Table 3:** Number of red deer teeth by age and sex.

Age (year)	2	3	4	5	6	7	8	9	10	11	12	13	14	15	16	17
female	7	6	6	7	6	4	7	5	5	6	3	6	5	6	6	1
male	10	5	4	6	5	5	5	4	5	6	2	5	6	2	0	0

### Tooth measures

**Fig 1:**
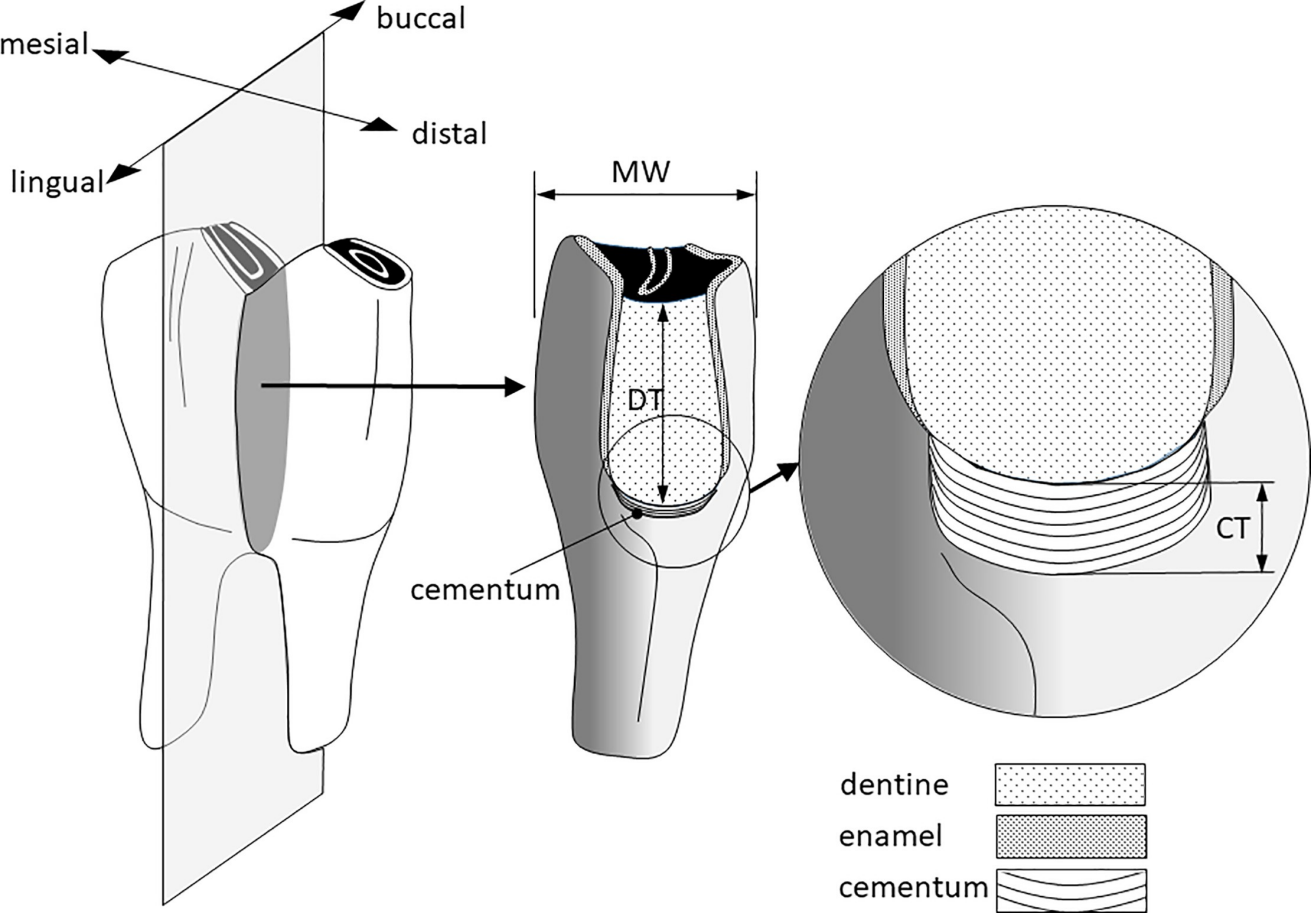
Red deer lower first molar section through its lingual-labial coronal plane. MW: maximum molar width (in mm); DT: dentine thickness (in mm); CT: cementum thickness (in mm). The figure is not at scale to improve visualisation.

**Fig 2:**
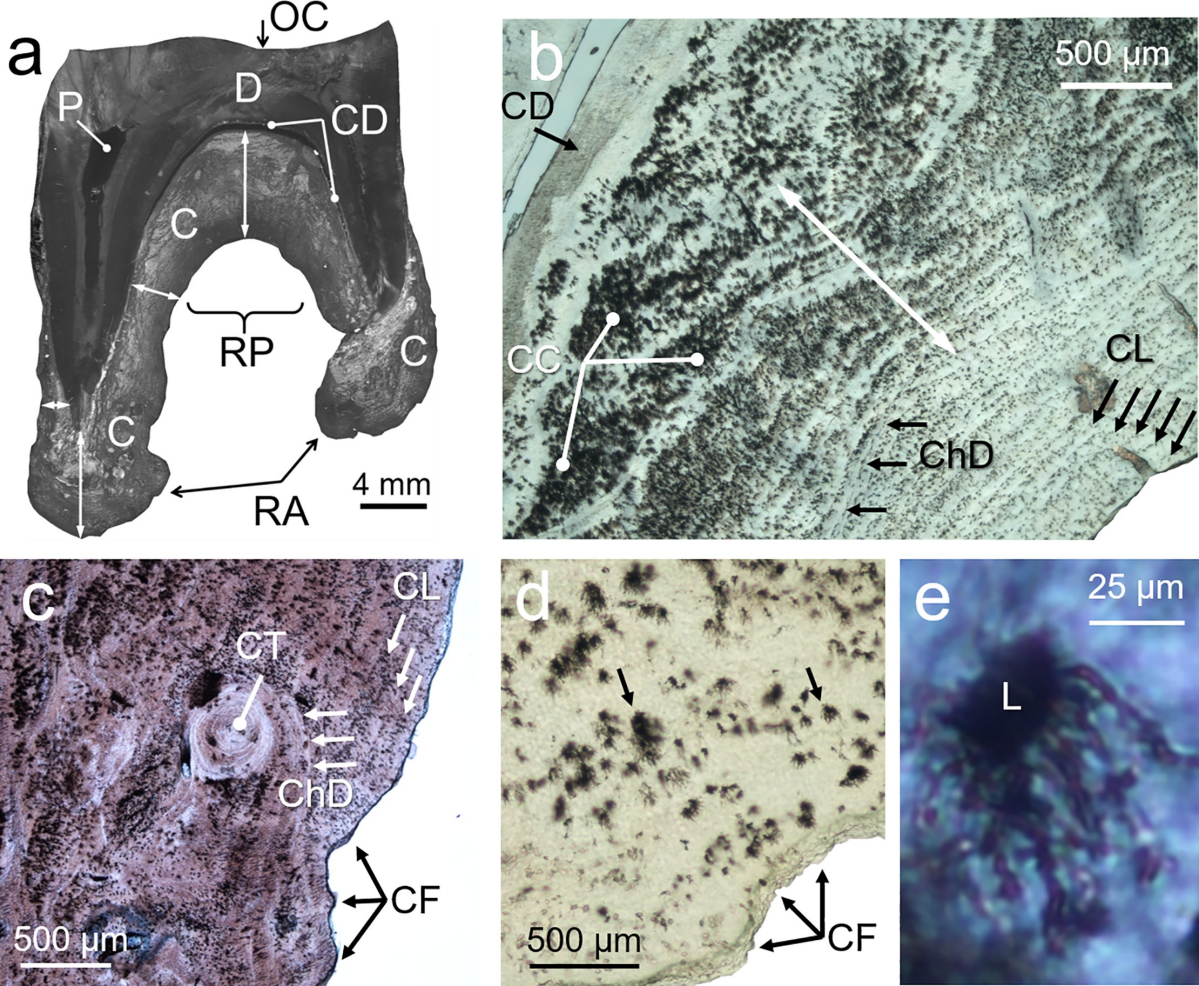
a. Sagital section of a first lower molar of a red deer hind (thickness: 350 μm). Occlusal surface (OC); dentine (D); pulp (P); cementum (C); dentine-cementum junction (CD); cementum radicular pad (RP); root apex (RA); double-headed arrows show variation in cementum thickness across different parts; cementum and dentine have become separated along CD at the top of RP. b. sagittal section of radicular cementum showing the direction of proliferation of cementum layers (white double-headed arrow); most recent cementum layers (CL); change of direction of cementum deposition (ChD); aggregation of cementocites (CC). c. as inset b showing multiple changes in direction (ChD) and different shapes of cementum deposition layers (CT); undulated cementum deposition front (CF). d. arrows show two cementocite lacunae; note that the direction of lacuna canaliculi proliferates in the same direction that cementum deposition does. e. detail of a cementocyte lacuna with its canaliculi expanding in the same direction of cementum deposition. Thickness of sections b-e was 70 μm. Section c was Von Kossa stained, all other sections were unstained.

Five traits were measured from each molar, (1) cementum thickness (in mm), as the depth of the cementum along the vertical axis of a buccal-lingual section of M_1_ (Fig 1); (2) dentine thickness (in mm), as a proxy of tooth wear, measured as the depth of the dentine along the vertical axis from the dentine-cementum junction of the radicular pad to the middle point of the occlusal plane on a buccal-lingual sectioned crown of M_1_ [8–10,27]; (3) molar width (in mm) as the maximum buccal-lingual width of M_1_, this was used in the analysis as a covariate to account for the size of the molar and size of the animal (see Statistics); and enamel and dentine micro-hardness in mega Pascal (MPa), measured on the polished surface of the buccal-lingual section of M_1_ by micro-indentation using a Vickers hardness tester (IndentaMet series 11100, Buehler), as described in Pérez-Barbería [27]. Although in red deer enamel is about four times harder than dentine [27], both traits were considered as both constitute the occlusal surface of the tooth and consequently tooth crown depletion will be affected by the combined hardness of enamel and dentine [3,27,28]. Special care was taken to measure tooth hardness on the same anatomical part of the enamel and normodentine [ie. primary dentine, the most homogeneous and mineralized type of dentine of the tooth [29]] to minimise variation between the five micro-hardness tests carried out within each tooth and across different animals. The mean of these five hardness tests was used in the statistical modelling. Micro-indentation measures the resistance of a material to plastic deformation [30], and although deformation is not the only process that operates during abrasion and attrition which affects wear [28,31], it has been effectively used as a proxy of tooth resilience to wear [27].

The reason why cementum thickness was measured as the thickness of all layers of cementum, rather than measuring the thickness of each annual layer, was the difficulty in identifying individual layers. Pérez-Barbería et al [13] found, in the same molar sample used in this study, that 50% of the estimates of age by counting cementum layers were under an error of +/- 1 year, and in 2% of the molars there was a bias up to 5 years younger and 4 years older than the actual age, which clearly indicate the poor definition of the boundary between successive layers in some molars.

All length measures were carried out using ImageJ free software [32] on digitalised images of polished sections of M_1_, which were digitalised on a flatbed scanner (Epson Perfection V39) at 4800 ppi. Further details in Pérez-Barbería [27].

Finally, thin histological M_1_ sagittal plane sections were prepared using the poly methyl methacrylate technique [33] to provide a graphical description of the layering pattern of the cementum radicular pad. The technique can be summarised as follows, (i) tooth samples are fixed in ethanol and washed in acetone and xylene, (ii) infiltrated in a solution of methyl methacrylate, benzoyl peroxide and poly methyl methacrylate, (iii) submerged in a clean solution described in point (ii), which is slowly polymerised under exposure to black light to create a hard block, (iv) thin sections of the block are prepared using metallographic grinding and polishing equipment.

### Statistics

We modelled root furcation cementum pad thickness in relation to age, sex, tooth size and wear, and dentine and enamel micro-hardness to test hypotheses H1 to H8. Molar width was included in the model (i) as a covariate that enables using dentine thickness as an inverse measure of the dentine that has been depleted over time and therefore a measure of tooth wear [22,27]; (ii) larger-bodied adults are normally larger at birth and their rates of growth are also faster than smaller conspecifics, especially before weaning [34,35]. Consequently, molar width can be used as a proxy of body size when it is fitted in the model together with age, and is normally not affected by tooth wear when it is measured at its maximum crown width [22]. Enamel and dentine micro-hardness were fitted in two separate models. The period between an animal´s death and the micro-hardness test (between 8 to 24 years) might affect tooth-hardness because of enamel or dentine degradation. However, using the same sample of teeth as in the present study, Pérez-Barbería [27] demonstrated that this potential effect was negligible and therefore it was not included in our models. In addition, we fitted cohort as a random effect in the model to account for any environmental or population conditions associated with the year of birth that could directly affect cementum formation, dentinogenesis or amelogenesis in the offspring or via maternal effects [27].

In an early part of the analysis we used generalised additive models GAM [36] in the R software package mgcv [37] to check for a potential non-linear response between dependent and explanatory variables. This was relevant because some hypotheses are based on changes in the rate of cementum deposition through life. Although GAM models are additive by definition, we fitted some pertinent interactions between explanatory variables, using the ti function within GAM model formulae, that produce a tensor product interaction which is appropriate when the main effects and any lower interactions are also present [38,39]. A thin plate regression spline with a penalized smoothing basis was used for fitting fixed-effects continuous variables, and random effects were treated as smooths (using the “re” basis in model formulae).The terms produce a parametric interaction of the predictors and penalize the corresponding coefficients with a multiple of the identity matrix, corresponding to an assumption of normality [39]. We tried a linear versus basis of modest size (*k* = 3 and *k* = 4) in the main effects and interactions. Model selection was performed using Akaike (AIC) weights aided by the normalised probability of the Kullback–Leibler discrepancy ratio, in which model A is to be preferred over competing model B [40]. The most parsimonious models were those that fitted linear responses, consequently, we used a linear mixed model approach implemented by the R software package lme4 [41] and lmerTest [42]. lmerTest provides p-values for models fitted using lme4 with Satterthwaite's degrees of freedom approximation, as in linear mixed-effects models determining the “correct” value of degrees of freedom in the estimate of the coefficients is meaningless [43,44]. The coefficients of the linear mixed model were calculated using restricted maximum likelihood REML, as the estimates are more accurate than using maximum likelihood [44]. The variance explained by the linear mixed model was represented as *R^2^* marginal (variance accounted for by the fixed effects (*R^2^*_LMM(m)_) and *R^2^* conditional (variance accounted for by random and fixed effects; *R^2^*_LMM(c)_), following a method developed for linear mixed-effects models [44] and used in different studies [10,22,27]. Graphics were created using the ggplot2 R package based on The Grammar of Graphics [45].

## Results

There was visual evidence that the cementum deposition rate was not constant across different parts of the molar (Fig 2A). The thickest layers of cementum were deposited between the roots and on their apexes, and the thinnest by the cementum-enamel junction. The cementum deposition front showed clear macroscopic indentations, mainly around the root apex, facilitating the anchorage of the tooth into the alveolar bone via the periodontal ligament. Fine undulations on the cementum deposition front were observed at greater magnifications, these undulations did not necessary match in shape and thickness across successive layers of cementum (Fig 2C and 2D). The pattern of deposition of cementum was highly variable. Although a layering pattern was predominant there were places where different fronts of deposition seemed to converge, some other places developed very sinuous layers and even concentric cementum nodules (Fig 2C). The canaliculi of the lacunae had more development in the direction of the cementum deposition (Fig 2D and 2E). The lacunae did not have a uniform spatial distribution, in some places they were aligned along cementum layers, in other places formed dense clusters that seems to span many layers of cementum (Fig 2B).

**Fig 3:**
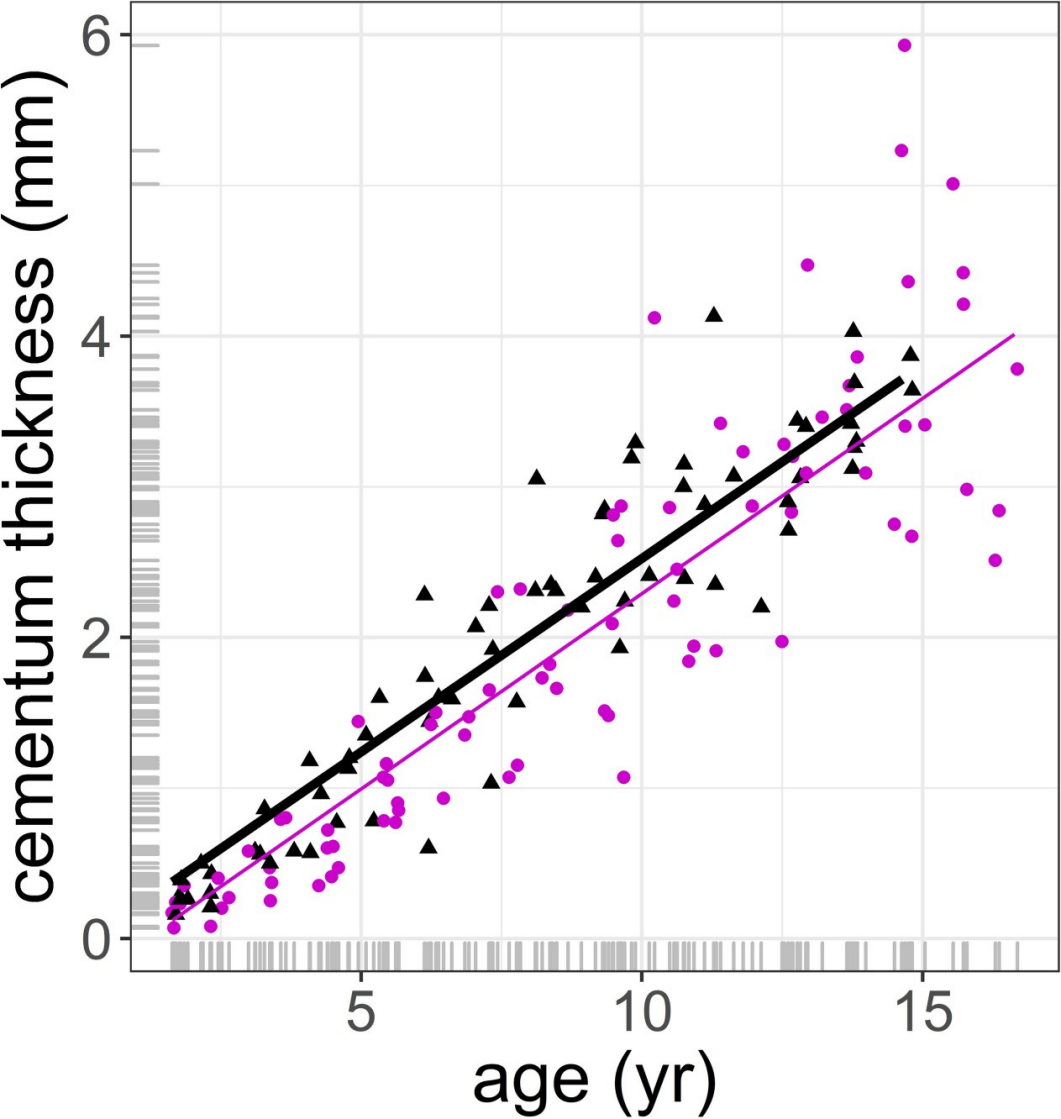
Prediction of inter-radicular cementum thickness pad (in mm) against age (in years) using the model in Table 4. Circle and purple thin line: female; triangle and black thick line: male. No significant differences in slope and intercept between sexes. The marginal distribution of x and y variables are displayed as grey ticks along the axes.

**Fig 4:**
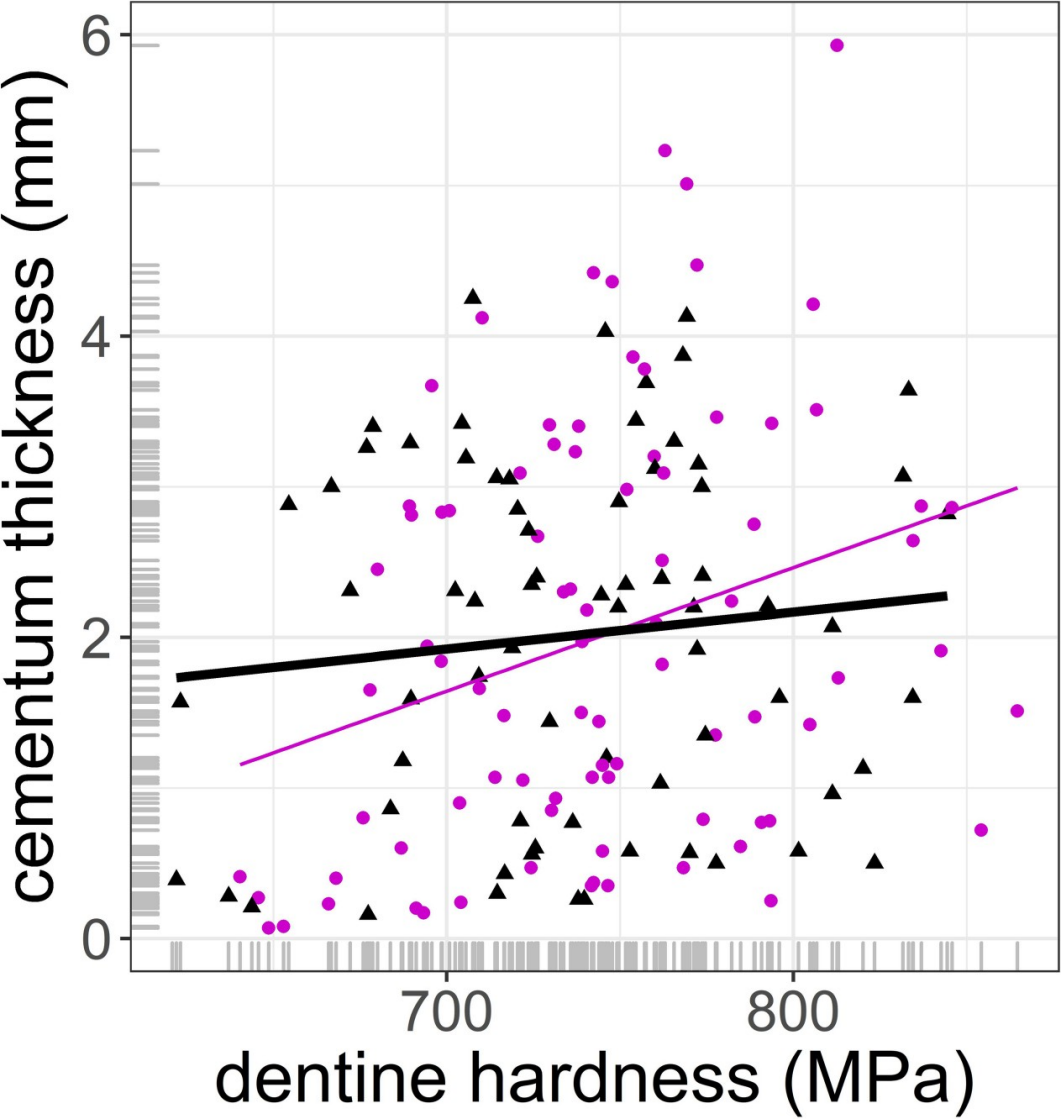
Prediction of inter-radicular cementum thickness pad (in mm) against dentine micro-hardness (in mega Pascal) using the model in Table 5. Circle and purple thin line: female; triangle and black thick line: male. No significant differences in slope and intercept between sexes. Details in Table 2.

After controlling for tooth size, cementum thickness increased with age at an annual rate of 0.26 mm (se = 0.024, p < 0.001), with no significant differences between sexes (Table 4, Fig 3). Dentine thickness had no effect on cementum thickness, which clearly indicates that cementum deposition is independent of tooth wear, other than that associated with age (estimate = -0.019 mm, se = 0.032, p = 0.553, Table 3). Neither enamel hardness (estimate = -3.16E-05, se = 3.08E-04, p = 0.918, Table 4) nor cohort (p < 0.001, Table 4) affected cementum thickness. The marginal and conditional *R^2^* explained 83% of the variance of the data. Similar results were obtained from a model that replaced enamel hardness with dentine hardness (S1 Table). An alternative model for cementum thickness that fitted only dentine hardness, sex and its interaction as fixed effects suggested that cementum thickness increased with dentine hardness (estimate = 0.004 mm, se = 0.002, p = 0.042, Table 5, Fig 4). However, this effect was subrogated by age when it was included in the model (Table 4). The model in Table 4 explained 2% of the marginal variance of the system and 58% of the conditional variance. A similar model to that in Table 5, in which dentine hardness was replaced with enamel hardness, showed no significant fixed effects (Supplementary material, Table 3).

**Table 4:** Coefficients of a linear mixed model on the inter-radicular cementum thickness pad (in mm), controlling for sex, molar size (molar width, MW, in mm), age (in years), dentine thickness (DT, in mm), enamel micro-hardness (EH, in MPa) and the pertinent interactions as fixed effects, and cohort as random effect. p (> Chi^2^): probability of tests of random-effect terms in the model, each term is removed and REML-likelihood ratio tests computed. *R^2^*_LMM(m)_ marginal variance accounted for the fixed effects; *R^2^*_LMM(c)_ conditional variance accounted for random and fixed effects.

Random effects	variance	sdev	p (> Chi^2^)		
cohort (n = 24)	0	0	1.0		
residual (n = 151)	0.292	0.540			
Fixed effects	estimate	se	df	t value	p
(Intercept)	1.389	1.246	143	1.115	0.267
MW	-0.142	0.068	143	-2.093	0.038
age	0.261	0.024	143	10.847	< 0.001
sex (male)	-0.044	1.554	143	-0.028	0.977
DT	-0.019	0.032	143	-0.594	0.553
EH	-3.16E-05	3.08E-04	143	-0.103	0.918
age × sex (male)	-0.007	0.022	143	-0.313	0.755
EH × sex (male)	1.06E-04	4.92E-04	143	0.216	0.830
*R^2^*_LMM(m)_	0.827				
*R^2^*_LMM(c)_	0.827				

**Table 5:** Coefficients of a linear mixed model on the inter-radicular cementum thickness pad (in mm), controlling for sex, dentine micro-hardness (DH, in MPa) and the interaction as fixed effects, and cohort as random effect. Details in Table 4.

Random effects	variance	sdev	p (> Chi^2^)		
cohort (n = 24)	0.998	0.999	< 0.001		
residual (n = 153)	0.755	0.869			
Fixed effects	estimate	se	df	t value	p
(Intercept)	-1.176	1.582	140.7	-0.744	0.458
DH	0.004	0.002	137.7	2.051	0.042
sex (male)	2.236	2.215	133.5	1.009	0.315
DH × sex (male)	-0.003	0.003	133.3	-1.051	0.295
*R^2^*_LMM(m)_	0.017				
*R^2^*_LMM(c)_	0.576				

## Discussion

The results clearly indicate that cementum deposition takes place at a constant rate across the life of the animals of our sample, supporting hypothesis H1 that cementum is a tissue whose main function is maintaining opposing teeth in occlusion [3]. However, contrary to H2, teeth that presented higher rates of tooth wear, and consequently needed more cementum to maintain the tooth in occlusion, did not have thicker cementum inter-radicular pads in comparison to less worn teeth. Despite this study having used the same sample of teeth in Pérez-Barbería [27], this study found faster rates of tooth wear in males than in females after controlling for tooth size and micro-hardness [Fig 6 in Pérez-Barbería [27]]. However, the rates of cementum deposition did not differ between sexes, which is contrary to prediction H3 based on the disposable soma theory of sexual selection [17]. Also related to the disposable soma theory is hypothesis H4, which predicts that males should decrease somatic investment after their prime, time at which their reproductive success decreases. The results did not support prediction H4, as the rate of cementum deposition remained constant with age.

The fact that the thickest deposition of cementum takes place on the radicular pad and root apex in a perpendicular direction to the occlusal plane (Fig 2A) suggest that cementum deposition is a mechanism to bring a worn tooth in occlusion with its opposing tooth. The finding that the rate of deposition across an animal´s life was constant and the pattern of cementum layering deposition was quite irregular across years (as indicated by visual observation Fig 2 and the regression residuals Fig 3), suggest that the rate of cementum deposition can be tuned to particular events. These events might be related to periods of greater tooth wear (i.e. changes in diet and food intake) or for improving tooth anchorage in the alveolar bone. Furthermore, our rate of cementum apposition with age was calculated as the thickness of the accumulated layers of cementum across the animal´s life, as it was not possible to measure each layer of cementum because not all were well defined. This implies that no information was available on actual annual cementum apposition. This might explain why only prediction H1 of the “maintenance of tooth in occlusion” hypothesis was supported by our statistical analyses but not the associated H2 prediction. However, it is still surprising that our proxy of tooth wear (i.e. dentine thickness controlling for molar size) did not have any significant effect or at least a positive trend on cementum apposition as predicted. We based the H2 prediction on the functional concept described by Hillson [3] that tooth occlusal surfaces can be repositioned by remodelling the periodontal ligament fibres and creation of new fibres that are eventually entrapped by the growing cementum. Experimental support seems inconclusive, in rats, in which an opposing molar was extracted, the growth of cementum of these molars was less than that of the control contralateral molar that their occlusion was not modified [46]. In a similar experiment on rats carried out by Tsuchiya et al [47] M1 was extracted, as a consequence the adjacent M2 and M3 showed a significant distal drift and the proliferation of acellular cementum on the distal side of the root of the molar but not on its mesial side. It is difficult to compare the results of both experiments, because the directions of the forces exerted on the teeth of both experiments are different. Despite this, Tsuchiya et al [47] experiment provides some support to prediction H2 that cementum is implicated in the mechanism of tooth repositioning.

A non-linear rate of cementum deposition was expected, with a decrease after their prime at around 10 years of age, when males´ reproductive success declines in this population [18]. It is well known that males of deer species that are highly dimorphic in body size reduce their feeding activity during rut [48,49], which results in lower deposition rates and thinner layers of cementum [12,16,29]. The result of this process across a male´s reproductive life should be reflected in reduced cementum thickness, in comparison with that of females of the same age, but this hypothesis (H5) was not supported by the results. It is plausible that (i) compensatory mechanisms might be involved eg., extra feeding activity after rut as an attempt to recover body condition [50], which might accelerate tooth wear and so cementum deposition; (ii) as not all males of a population are equally involved in rut through their lives [51–53], it could be possible that this effect was negligible in many males and so difficult to detect across the population. Harder teeth should have a lower rate of tooth wear and in agreement with H2 these teeth should have lower rates of cementum deposition (H6). Contrary to the prediction of H6, a simple model that fitted dentine hardness, sex and its interaction revealed that cementum deposition increased with dentine hardness, but when age was included in the model the effect disappeared, as predicted in H7. This could be explained by the positive effect that age has on dentine and enamel mineralisation [26]. Pérez-Barbería [27] found that dentine and enamel micro-hardness increased with age up to 10 in red deer, probably due to the mineralisation process during maturation of these two tissues. It could be hypothesised that this age effect supersedes the expected negative effect of tooth hardness on cementum deposition. It is, however, unclear why the effect was detected on dentine hardness but not enamel hardness. It is plausible that this is because the coefficient of variation (CV) of enamel micro-hardness is more than twice that of dentine (enamel CV = 5.3%, dentine CV = 2.2%) [27], which makes it more difficult to detect small responses to variation in enamel hardness. The fact that the rate of deposition of cementum was constant through life does not support prediction H8, as our GAM explorative models did not detect any significant change in the rate of deposition at the age when males are expected to become competitive in reproduction; neither at the oldest ages when highly worn teeth might need extra support from the periodontal ligament. In relation to the latter, it is possible that the small sample size at the oldest ages made it more difficult to detect any change in the rate of deposition.

It could be hypothesised that rates of cementum deposition are faster in adult males than in females, as a mechanism of improving the attachment of the tooth to the alveolar bone during the processes of bone resorption that takes place during the phase of antler growth. During this period it has been claimed that a process of bone resorption (osteoporosis) takes place as insufficient mineral uptake can occur with a natural diet [54–57]. However, it seems unlikely that bone resorption has been under positive selection on the mandibular bone, which would put at risk the attachment of teeth, crucial structures for food intake and digestion. Nevertheless, our results do not support this hypothesis, as significant differences were not found between the sexes in the intercepts or in the slopes of the regression lines of cementum thickness versus age after controlling for molar size.

We remark that our hypotheses and predictions are based on the biology and life history traits of deer species, concretely red deer, and therefore generalisations of our results on the cementum apposition patterns of other mammalian or vertebrate species should be considered with caution. To the best of our knowledge most of the studies carried out on cementum deposition have focused on temporal apposition patterns to detect chronological and physiological events. More work has been carried out on dentine. In belugas (Delphinapterus leucas) it was hypothesised that rates in dentine deposition changed at sexual maturity, but the results were inconclusive [58]. In humans, circadian rhythms of dentine apposition have been identified using fluorochrome markers, but no information on deposition rate changes were provided [59]. A follow up study by Dean and Scandrett [60], also in humans, found that rates of dentine formation varied in different parts of the tooth across a period of 40 months. In some parts the rate was constant while in other parts accelerated or slowed, apparently in order to match the rates of growth of the different parts of the tooth. The use of bone-labeling technique is a useful tool to track dynamics of bone formation and remodelling under experimental conditions and when animals can be handled [61]. However, the technique is logistically intractable to be applied to wild ungulates on longitudinal population studies to assess hypotheses on life history traits. As a compromise, and to the best of our knowledge, our study is the first to use population cross-sectional cementum thickness data and statistical modelling approach to assess functional and evolutionary hypotheses on cementum apposition rates in a mammalian species.

In conclusion, cementum deposition in red deer takes place at a constant rate across an animal's life with no differences between the sexes, even after controlling for tooth size. The rates of cementum deposition do not respond as expected to any of the sexual selection hypotheses stated in this study, which are related to the highly polygynous mating strategy of this species. Our results suggest that cementum´s main function is to maintain precise positioning of the tooth occlusal surface as a response to changes in the occlusal surface and alveolus associated with age. We, however, could not support the prediction that once the contribution of this effect was accounted for cementum apposition responded positively to tooth wear. Lack of detailed information on cementum apposition rates across a variety of mammalian and reptilian species make it difficult to identify a single evolutionary mechanism that might have driven the patterns of cementum deposition.

## Supporting information

S1 Table (DOCX)

S2 Table (DOCX)
