# Supplementary material for: What do rates of deposition of dental cementum tell us? Functional and evolutionary hypotheses in red deer
Source: PLoS One. 2020 Apr 28;15(4):e0231957. doi: 10.1371/journal.pone.0231957 (PMC7188284; doi:10.1371/journal.pone.0231957)
Supplement: S1 Table — (DOCX) [file pone.0231957.s001.docx]

Supporting information Table 1. Coefficients of a linear mixed model on the inter-radicular cementum thickness pad (in mm), controlling for sex, molar size (molar width, MW, in mm), age (in years), dentine thickness (DT, in mm), dentine micro-hardness (DH, in MPa) and the pertinent interactions as fixed effects, and cohort as random effect. p (> Chi^2^): probability of tests of random-effect terms in the model, each term is removed and REML-likelihood ratio tests computed. *R^2^*_LMM(m)_ marginal variance accounted for the fixed effects; *R^2^*_LMM(c)_ conditional variance accounted for random and fixed effects.

| Random effects | variance | sdev | p (> Chi^2^) |  |  |
| --- | --- | --- | --- | --- | --- |
| cohort (n = 24) | 0 | 0 | 1.0 |  |  |
| residual (n = 151) | 0.290 | 0.538 |  |  |  |
| Fixed effects | estimate | se | df | t value | p |
| (Intercept) | 0.397 | 1.221 | 143.0 | 0.325 | 0.746 |
| MW | -0.140 | 0.068 | 143.0 | -2.071 | 0.040 |
| age | 0.259 | 0.024 | 143.0 | 10.831 | < 0.001 |
| sex (male) | 1.166 | 1.314 | 143.0 | 0.888 | 0.376 |
| DT | -0.016 | 0.032 | 143.0 | -0.498 | 0.620 |
| DH | 0.001 | 0.001 | 143.0 | 0.945 | 0.346 |
| age × sex (male) | -0.003 | 0.023 | 143.0 | -0.112 | 0.911 |
| EH × sex (male) | -0.001 | 0.002 | 143.0 | -0.68 | 0.497 |
| *R^2^* _LMM(m)_ | 0.828 |  |  |  |  |
| *R^2^* _LMM(c)_ | 0.828 |  |  |  |  |
