# Supplementary material for: What do rates of deposition of dental cementum tell us? Functional and evolutionary hypotheses in red deer
Source: PLoS One. 2020 Apr 28;15(4):e0231957. doi: 10.1371/journal.pone.0231957 (PMC7188284; doi:10.1371/journal.pone.0231957)
Supplement: S2 Table — (DOCX) [file pone.0231957.s002.docx]

Supporting information Table 2. Coefficients of a linear mixed model on the inter-radicular cementum thickness pad (in mm), controlling for sex, dentine micro-hardness (EH, in MPa) and the interaction as fixed effects, and cohort as random effect. Details in Table 4.

| Random effects | variance | sdev | p (> Chi^2^) |  |  |
| --- | --- | --- | --- | --- | --- |
| cohort (n = 24) | 1.101 | 1.050 | < 0.001 |  |  |
| residual (n = 153) | 0.768 | 0.877 |  |  |  |
| Fixed effects | estimate | se | df | t value | p |
| (Intercept) | 1.750 | 1.731 | 136.9 | 1.011 | 0.314 |
| EH | 9.02E-05 | 5.48E-04 | 133.3 | 0.165 | 0.870 |
| sex (male) | 0.643 | 2.688 | 130.6 | 0.239 | 0.811 |
| EH × sex (male) | -2.38E-04 | 8.48E-04 | 130.5 | -0.281 | 0.779 |
| *R^2^* _LMM(m)_ | 0.002 |  |  |  |  |
| *R^2^* _LMM(c)_ | 0.590 |  |  |  |  |
